# HPV in HIV-Infected Women: Implications for Primary Prevention

**DOI:** 10.3389/fonc.2014.00179

**Published:** 2014-08-12

**Authors:** Nathalie Dauphin McKenzie, Erin N. Kobetz, Parvin Ganjei-Azar, Isabella Rosa-Cunha, JoNell E. Potter, Atsushi Morishita, Joseph A. Lucci, Toumy Guettouche, James H. Hnatyszyn, Tulay Koru-Sengul

**Affiliations:** ^1^Division of Gynecologic Oncology, Department of Obstetrics and Gynecology, University of Miami Miller School of Medicine, Miami, FL, USA; ^2^Department of Public Health Sciences, University of Miami Miller School of Medicine, Miami, FL, USA; ^3^Division of Gynecologic Oncology, University of Florida Health Cancer Center – Orlando Health, Orlando, FL, USA; ^4^Sylvester Comprehensive Cancer Center, University of Miami Miller School of Medicine, Miami, FL, USA; ^5^Department of Pathology, University of Miami Miller School of Medicine, Miami, FL, USA; ^6^Division of Infectious Diseases, Department of Medicine, University of Miami Miller School of Medicine, Miami, FL, USA; ^7^Division of Women’s Health Research, Department of Obstetrics and Gynecology, University of Miami Miller School of Medicine, Miami, FL, USA; ^8^Bio-Medical Department, Kurabo Industries LTD, Osaka, Japan; ^9^Oncogenomics Core Facility, Sylvester Comprehensive Cancer Center, University of Miami Miller School of Medicine, Miami, FL, USA; ^10^Division of Hematology and Oncology, Department of Medicine, University of Miami Miller School of Medicine, Miami, FL, USA

**Keywords:** HPV, HIV, CIN, cervical cancer

## Abstract

**Background:** There is growing evidence that human immunodeficiency virus (HIV)-infected women might have a different human papillomavirus (HPV) type distribution in cervical dysplasia specimens as compared to the general population. This has implications for primary prevention.

**Objective:** We aimed to obtain preliminary data on the HPV genotypes prevalent in histological samples of HIV-infected women with cervical intraepithelial neoplasia (CIN) 3/CIS of the cervix in Miami, FL, USA.

**Methods:** Retrospective data were collected on HIV-infected women referred to the University of Miami-Jackson Memorial Hospital colposcopy clinic between years 2000 and 2008. The histology slides of CIN 3/CIS biopsies underwent pathological review and sections were cut from these archived specimens for HPV DNA extraction. HPV genotyping was then performed using the GeneSquare™ HPV genotyping assay. We report on our first set of 23 samples.

**Results:** Eight high-risk HPV types were detected. Types in decreasing order of frequency were 16, 35, 45, 52, 59, 31, 58, and 56. Most cases had multiple infections. HPV type 16 was the most common (45%) followed by HPV-35 and -45 with equal frequency (40%). No samples contained HPV-18.

**Conclusion:** Our preliminary results suggest that cervical dysplasia specimens of HIV-infected women more likely (55%) contain non-16 and -18 high-risk HPV types. We show that this held true for histologically confirmed severe dysplasia and carcinoma-*in situ*. Epidemiological studies guide vaccine development, therefore HPV type prevalence in CIS and invasive cervical cancer among HIV-infected women should be more rigorously explored to ensure that this highly vulnerable population receives appropriate primary prevention.

## Introduction

Epidemiological studies demonstrate that high-risk human papillomavirus (HR-HPV) genotypes are the necessary cause of 99.7% of cervical cancer. Seven of them [human papillomavirus (HPV)-16, -18, 45, 31, 33, 52, 58] account for nearly 90% of all cervical cancer cases in the general population worldwide with little regional variation (1). HPV types 16 and 18 together account for the great majority (70%) of cervical carcinomas in the general population. HPV-16 and -18 are far less common in low grade pre-cancerous lesions. Rather, their prevalence increases with grade of cervical dysplasia. However, there is growing evidence that human immunodeficiency virus (HIV)-infected women might have a different HPV type distribution in cervical dysplasia specimens as compared to the general population (2). When HIV-infected women are infected with HR-HPV, the natural history of the HPV infection and subsequent carcinogenesis is different when compared to non-HIV-infected women. Specifically, HIV-infected women with HR-HPV progress more rapidly to high-grade dysplasia and invasive cervical cancer; the dysplasia is more likely to recur after treatment; and when invasive cancer does develop it responds poorly to standard treatment (2). Because extensive epidemiological studies were instrumental in determining the HR-HPV types commonly found in invasive cervical cancer thereby guiding the development of the current HPV vaccines, it would be equally important to perform the same rigorous epidemiological investigation for HIV-infected women. The two FDA-approved HPV vaccines protect against HR-HPV-16 and 18 while one of them also protects again HPV types 6 and 11 which are not oncogenic but are associated with genital warts. As previously mentioned, HIV-infected women with abnormal cervical cytology specimens tend to be infected with non-16 and -18 HR-HPV types, such as 51, 52, 53, 56, and 58, but the most prevalent type appeared to differ by geographic region (Table 1) (2). One criticism of these studies on HIV-infected women is that the samples contained little or no invasive cancer cases. Also, only cytological (Pap smear) diagnoses of low grade intraepithelial lesions (LGSIL) or rarely, high-grade squamous intraepithelial lesions (HGSIL) were obtained. HGSIL is a cytological diagnosis, which groups together moderate and severe dysplasia. To have clinical relevance, all cytology results must be confirmed through biopsies to obtain a histological diagnosis of grade of dysplasia or cervical intraepithelial neoplasia (CIN). These diagnoses are defined as follows: CIN 1 (mild dysplasia), CIN 2 (moderate dysplasia), and CIN 3 (severe dysplasia and carcinoma-*in situ*). This histological confirmation was not performed in previously reported studies on HPV type prevalence in HIV-infected women (2).

**Table 1:** **Common HPV genotypes in HIV-infected women – summary (2)**.

Study^c^	HPV types more common than 16
Palefsky et al. (3)	53^b^, 58
Levi et al. (4)	51, 18
Levi et al. (5)	^a^
La Ruche et al. (6)	^a^
Luque et al. (7)	56, 53^b^
Sahasrabuddhe et al. (8)	52, 58

*^a^16 was most prevalent in this study*.

*^b^HPV type 53 is considered probably carcinogenic by the Munoz (1) classification*.

*^c^References from Ref. (3–8)*.

There is sufficient clinical trial data to show that there is modest cross-protection by the current HPV-bivalent (16/18) and quadrivalent (6/11/16/18) vaccines against additional HR-HPV types (9–11). The L1 protein, a late gene product of the HPV, can auto-assemble into an empty (non-DNA containing) virus like particle (VLP). These L1 VLPs are morphologically close to authentic HPV virions except that they lack the viral genome. VLPs possess the conformational epitopes required for the induction of neutralizing antibodies. Specifically, the antibody responses are typically generated against epitopes found on the external loops of the L1 protein present on the outer VLP surface (12). Protection against HPV requires the full-length protein (intact VLP). Protection is also HPV type-specific with the L1 VLPs. L2, a minor capsid protein, was also investigated as a vaccine candidate and L2-VLPs were found to be less efficient than L1, but were able to induce HPV type cross-protective antibodies (13). This may in part relate to differing L1 epitopes of different HPV types. For example, the amino-acid sequence of HPV-16 L1 shares greatest homology with HPV-31 L1 and HPV-35 L1 than with HPV-33 L1 (83.1, 82.8, and 79.7% amino-acid sequence identity, respectively) (14). However, because neutralizing epitopes of HPVs are conformation-dependent, their amino-acid composition and surface localization have not been fully characterized. Work by Combita et al. (15) showed that linear epitopes within the L1 protein could induce cross-neutralization; however, the cross-neutralization induced by L1 VLPs represents <1% of the neutralizing activity induced by dominant conformational epitopes, which is unlikely to confer sufficient cross-protection *in vivo*. With the overwhelming evidence that the current HPV vaccines cannot offer sufficient cross-protection with many other HR-HPV types, it is imperative that the most common types associated with malignancy in HIV, after HPV-16 and -18 be determined. Also, given that HIV-infected women are at greatest risk of developing HPV-related disease and dying from cervical cancer, it would be important to know whether the HPV type distribution in this patient population is similar to that of the general population thereby justifying the use of current HPV vaccines in HIV-infected women. Furthermore, the Hepatitis B vaccine trials showed that HIV-infected women do benefit from vaccines against oncogenic viruses with a 50–55% sero-conversion rate. The vaccine is more efficacious in individuals with higher CD4 cell count and low HIV viral loads (16). Nevertheless, Hepatitis B vaccines are recommended for HIV-infected individuals and if the HPV vaccine proves to be effective in this population, we can expect that the vaccine will become part of the vaccination protocol for this group as well.

**Figure 1:**
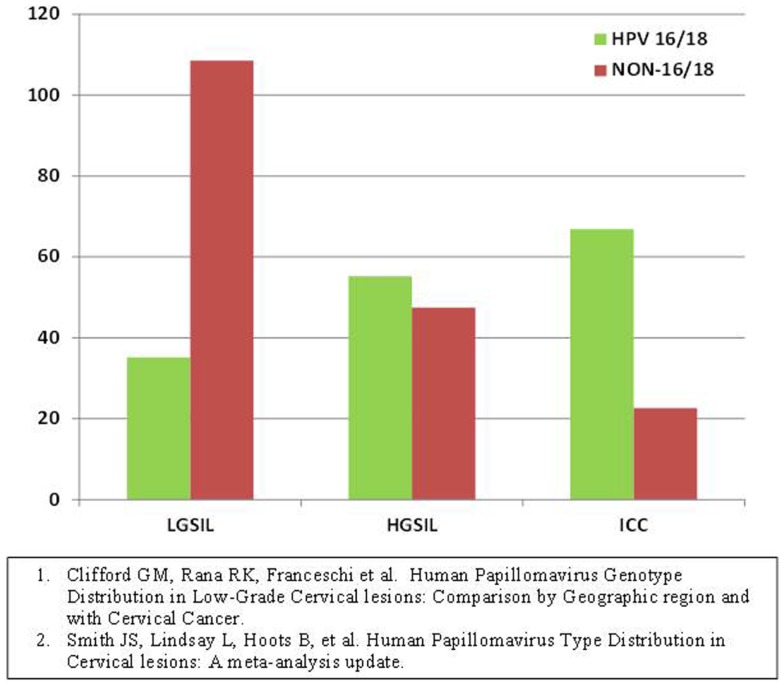
**Human papillomavirus type prevalence in different grades of dysplasia pooled estimates from all continents, general population (22, 23)**.

Meta-analyses have shown that, in the general population, many HR-HPV types are common in low grade cervical dysplasias however, as the grade of dysplasia increases HPV types 16 and 18 predominate. This follows a fairly linear upward trend. Similarly, as the grade of dysplasia increases, the other HR-HPV types become less common following a downward trend (Figure 1). High-grade dysplasia or CIN 3 is the most immediate pre-cursor to invasive cervical cancer. The HPV types found in CIN 3 are similar to those found in cervical cancer. Furthermore, CIN 2 and lesser grade lesions have poor diagnostic reproducibility and have less positive predictive value than CIN 3 (17). Our aim was to obtain histological specimens with diagnosis of CIN 3 from HIV-infected women for HPV genotyping with the ultimate goal of providing preliminary evidence on the types of HR-HPV associated with malignant transformation in this population.

## Materials and Methods

**Figure 2:**
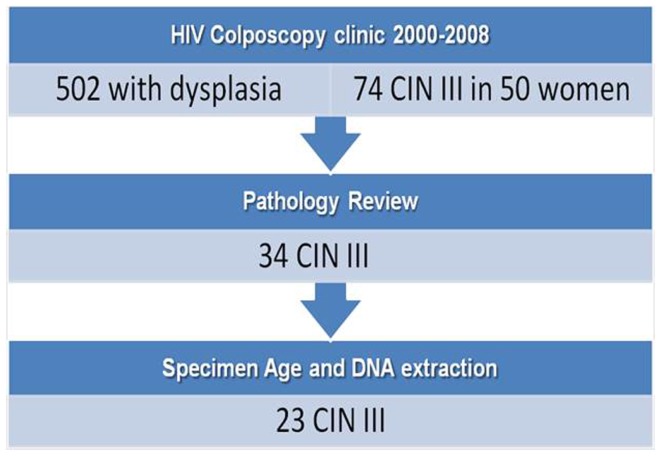
**Selection flowchart**.

After obtaining IRB consent, retrospective de-identified clinical data were collected on 510 HIV-infected women with abnormal Pap smears seen at the University of Miami-Jackson Memorial Hospital (UM-JMH) colposcopy clinic between 2000 and 2008 (Figure 2). This clinic evaluates women by performing Pap smears, colposcopies, and biopsies (when visual abnormalities are found) of the cervix, vulva, and anus every 6 months until they have normal colposcopic and histological results for at least 1 year or undergo surgical treatment for a high-grade pre-cancerous lesion. Because HIV-infected women under our care are followed closely (every 6 months) with regards to HPV-related disease, those that are compliant with treatment rarely progress to invasive cervical cancer. In fact, those with cervical cancer in the database were well known for their non-compliance with follow-up or were new patients with a pre-existing cervical cancer. Therefore, because of the rarity with which we see invasive cervical cancer in the patients followed at the UM-JMH colposcopy clinic and the fact that CIN 3 is considered to have positive predictive value, we decided to obtain genotyping data on CIN 3 specimens from these women to allow for a larger sample size than would have been possible by targeting invasive cancers.

Fifty patients (74 events) were diagnosed with CIN 3 (severe dysplasia/carcinoma-*in situ*) by cervical biopsies. Twenty patients had samples that were considered probably too old for successful DNA extraction and were excluded. Samples were limited to one event per patient using the case with the most severe pathology. All slides underwent central Gynecologic Pathology review. There were 24 of 30 remaining histology slides that were confirmed CIN 3 while the others were down-graded to CIN 2. The corresponding paraffin-embedded blocks of the CIN 3 were pulled and two 20-micron sections were cut from each block. DNA was extracted from these sections using the Ambion™ Recover All kit as per manufacturer’s instructions. We were able to successfully extract DNA for HPV genotyping on 23 samples. Clinical data on the final 23 cases were also obtained including patient demographics, cytological/histological results, as well as CD4 counts and HIV-1 viral load. After DNA was extracted from paraffin sections, we performed Nanodrop analysis to check concentration/ratio of DNA. This was followed by analysis of DNA on an Agilent™ Bioanalyzer chip to assess quality. HPV genotyping of these samples was performed using the Genesquare™ kit developed by Kurabo Industries LTD (Bio-medical Department, Kurabo Industries LTD., Osaka, Japan), which tests for 23 HPV types including all high-risk types and some low risk types. This kit uses multiplex PCR assays with HPV type-specific primers. We chose this PCR-sequencing method because it offers sensitive detection of HPV types at a lower cost and with increased processing speed than the standard Roche Linear array. Furthermore, there is no cross-hybridization because HPV type-specific primers are used as compared to the Roche method, which uses the L1 consensus primer (high-homology region). However, the Genesquare assay has not yet been validated in a large trial.

## Statistical Methods

De-identified study data were stored and managed in Microsoft Excel. SAS version 9.2 for windows (SAS Institute Inc., Cary, NC, USA) was used for statistical analysis. Descriptive statistics such as mean, standard deviation, median, first and third quantiles were reported for patients’ age and some of patients’ clinical characteristics, such as number of HPV infections, viral load, and CD4 count. Frequencies and percentages were calculated for characteristics such as race/ethnicity, grade of dysplasia, treatment, and recurrence of dysplasia. One-sample exact binomial test was used to test the differences in HPV genotype prevalence in cervical lesion of women between HIV-infected women and the general population reported. Type-I error rate is set to 5%.

## Results

**Figure 3:**
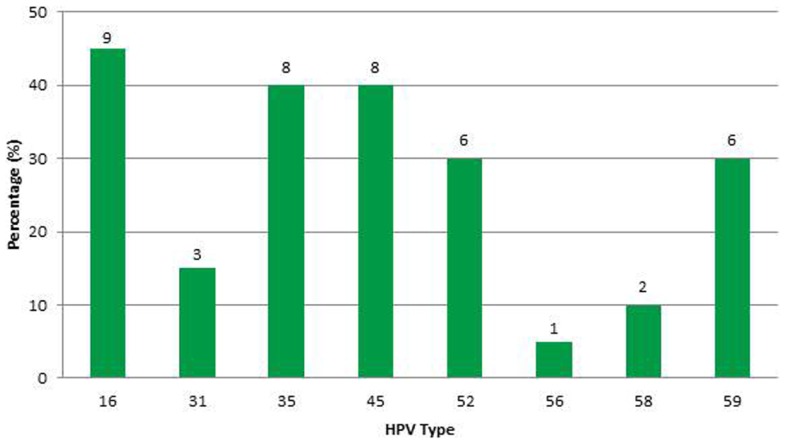
**Human papillomavirus genotype distribution (per infection) in HIV-infected women**.

In this sample (*n* = 23), 83% of women were African-American or Haitian-American and 50% were age 40 or younger (Table 2). Mean CD4 count was 193 (min; max: 15; 333). Mean Viral Load was 6,982 (min; max: 24; 639,000). Three samples had no detectable HPV DNA (or the DNA copies were below the assays’ threshold). Most cases had multiple infections with two to five high-risk types in the same sample and a total of 43 HPV infections in the remaining 20 samples. Eight HR-HPVs were detected. The types in decreasing order of frequency were 16, 35, 45, 52, 59, 31, 58, and 56 (Figure 3). Interestingly, HPV-16 was one of few types to stand alone (six HPV-16, one HPV-31, one HPV-59). HPV-16 was the most common (45%) per sample followed by HPV-35 and HPV-45 with equal frequency (40%). None of the samples contained HPV-18 (Table 3). All but one of the HR-HPVs detected were from the alpha-9 or alpha-7 species – the most carcinogenic among high-risk types (Table 4). Finally, 55% of the samples contained HR-HPV types other than -16 or -18. Specifically, HPV types 35, 45, 52, and 59 were significantly more prevalent in high-grade lesions of HIV-infected women in Miami, than in the high-grade lesions of women in the general populations (*p* < 0.05). However, the prevalence of HR-HPV types 16, 31, 56, and 58 were not significantly different than those in cervical lesions of women in the general population.

**Table 2:** **Patients’ characteristics (*n* = 23)**.

Characteristics	All, *n*(%)^a^
**Age**
Mean (StdDev)	37 (8)
Median (Q1; Q3)	40 (32; 41)
Min–max	18–52
**Race/Ethnicity**
African-American	14 (60.86)
Haitian	5 (21.74)
Latino	2 (8.70)
White	2 (8.70)
**Grade of dysplasia**
Severe dysplasia (CIN 3)	13 (56.52)
Carcinoma-*in situ* (CIS)	10 (43.48)
**HPV-16/-18**
HPV-16/-18	9 (45.00)
not HPV-16/-18	11 (55.00)
Not determined	3
**Viral load count**
Mean (StdDev)	6,982 (171,704)
Median (Q1; Q3)	2,344 (477; 24,700)
Min–max	24–639,000
Count ≤10,000	10 (62.50)
Count >10,000	6 (37.50)
**CD4 count**
Mean (StdDev)	193 (174)
Median(Q1; Q3)	146 (28; 315)
Min–max	15–533
Count ≥ 200	12 (60)
Count <200	8 (40)

*^a^*n* and percentages are presented unless otherwise specified; StdDev, standard deviation; Q1: 25th%; Q3: 75th%*.

**Table 3:** **Human papillomavirus types by grade of dysplasia**.

Patient ID	HPV-16/-18	HPV type	Grade of dysplasia
1	No	35, 56	SD
2	No	45, 52	CIS
3	Yes	16	SD
4	–	ND	CIS
5	Yes	16, 35, 45	SD
6	No	35, 45	CIS
7	No	59	SD
8	No	35, 59	SD
9	No	31	SD
10	No	35, 45, 52	CIS
11	No	31, 45, 52, 59	SD
12	No	35, 59	CIS
13	Yes	16	CIS
14	Yes	16, 35, 45, 52	CIS
15	No	45, 52, 58	SD
16	Yes	16	SD
17	Yes	16	SD
18	No	31, 58, 59	CIS
19	Yes	16	CIS
20	Yes	16	SD
21	–	ND	SD
22	Yes	16, 35, 45, 52, 59	CIS
23	–	ND	SD

*ND, non-detected (DNA viral copies below threshold for assay)*.

*SD, severe dysplasia*.

*CIS, carcinoma-in situ*.

**Table 4:** **Types by species and single infection**.

HPV type	Frequency (*n*, % per 20 samples)	Alpha species	*n*, % single infection
16	9, 45	9	6, 75
31	3, 15	9	1, 12.5
35	8, 40	9	0
45	8, 40	7	0
52	6, 30	9	0
58	2, 10	9	0
59	6, 30	7	1, 12.5
56	1, 5	6	0

## Discussion

In conclusion, we found that certain HR-HPV types that are considered relatively rare in high-grade cervical lesions of women in the general population were actually more common in high-grade lesions of HIV-infected women at the UM-JMH colposcopy clinic. We also found that the most prevalent HPV types (16, 35, 45) in these high-grade lesions among HIV-infected women at our institution differed from those of other HIV-infected women in other geographic regions (Table 5).

**Table 5:** **Human papillomavirus genotype prevalence (worldwide) in HGSIL compared to HIV+ in Miami**.

HPV genotype	HGSIL (%)^a^	HIV HGSIL(%)
16	45.3	45
18	6.9	–
45	2.3	40^c^
33	7.3	–
31	8.6	15
58	7.0	10
52	5.1	30^c^
35	3.8	40^c^
59	0.8	30^c^
53	–^b^	–
51	3.6	–
66	1.9	–
39	2.0	–
6	2.2	–
56	2.9	5

*^a^Smith JS, Lindsay L, Hoots B, et al. Human papillomavirus type distribution in cervical lesions: a meta-analysis update*.

*ICC: invasive cancer*.

*^b^The updated meta-analysis by Smith did not include type 53 in their publication of HGSIL unlike the previous publication by this group with regards to LGSIL and ICC*.

*^c^*p*-Value <0.05, which was calculated by one-sample exact binomial test for comparing our sample prevalence with the prevalence reported in (b) HGSIL and in (c) ICC. *P*-value <0.05 implies that the HPV genotype prevalence in cervical lesions of HIV-infected women in Miami, FL, USA is SIGNIFICANTLY different than the HPV genotype prevalence in cervical lesions of women in the general population. SIGNIFICANT HPV types are 35, 45, 52, and 59. NOT SIGNIFICANT HPV types are 16, 31, 56, and 58, i.e., *p*-value ≥0.05, i.e., the HPV genotype prevalence in cervical lesions of HIV-infected women in Miami, FL, USA is NOT SIGNIFICANTLY different than the HPV genotype prevalence in cervical lesions of women in the general population*.

Among the HPV types found in the HIV-infected population, HPV types 52, 53, 58, and 56 are the most commonly cited. In contrast, we found HPV-35, -16, and -45 to be the most common in our samples and none had HPV-18. This might be explained by the fact that all specimens were of squamous histology while HPV-18 is more commonly found in adenocarcinomas of the cervix (18).

In our sample, 95% (19/20) of the HPV types detected belonged to either alpha-9 or alpha-7 species. The alpha species of HPV are those subgroups of the virus that infect the genito-anal epithelium (19). The population-based results from Schiffman’s Guanacaste Project in Costa Rica (2005) demonstrate wide variability in HPV type-specific prevalence. They also found that the more likely a viral type was to persist, the more prevalent it was in the population (Spearman correlation coefficient 0.46, *p*-value = 0.005). In that same article, the most carcinogenic HPV types concentrated in species alpha-9 and alpha-7 were distinguished by elevated risk of progression given persistence, rather than persistence alone. Alpha-5 and alpha-6 species were also at elevated risk of progression given persistence (20). The fact that the majority of the HPV types found in CIN 3 of HIV-infected women in Miami, FL, USA belong to the alpha-9 and alpha-7 species further supports the findings of Schiffman and colleagues. Specifically, these authors reported that the alpha-9 and alpha-7 species were the most carcinogenic given persistence (20). The presence of highly carcinogenic HPV in our cervical samples of severe dysplasia and carcinoma-*in situ* is not surprising. Despite their high oncogenic potential, however, these other HPV types are less prevalent than HPV-16 and -18 in the general population. Nothing currently exists in the literature that explains why HIV-infected women are more susceptible to these typically less prevalent types. It is possible that despite the oncogenic potential of these other HPV types (35, 45, 52, and 59) they are not able to evade the immune system as efficiently as HPV types 16 and 18 except in HIV-infected individuals who are immunocompromised. To test this hypothesis regarding immunocompromised status and link with HPV type prevalence, a simple genotyping study in immunocompromised women not due to HIV (such as chronic steroid use, leukemia, renal transplant recipients) could be evaluated.

Usually HIV-infected women are infected with multiple types of HPV concurrently. The great majority of cases in our sample did in fact contain multiple HR-HPV types. One sample had as high as five concurrent HPV types simultaneously. While a few samples (*n* = 8) contained a single infection. HPV-16 accounted for 75% of these single infections (Table 4). These findings are both interesting and hypothesis generating. It is possible that the other HPV types act synergistically to promote carcinogenesis and are less efficient at carcinogenesis as single infections while HPV-16 is capable of inducing malignant transformation more efficiently than the others as a single infection. The activity of HPV oncoproteins E6 and E7 (largely responsible for malignant transformation of cervical cells) from different HPV types should be compared with regards to their likelihood of inducing carcinogenesis. Yet, another explanation is that the immune system is so compromised that it allows any pathogen to infect and persist within the human host as is seen with opportunistic infections in HIV-infected individuals. To test this hypothesis, we would expect to see the rate of multiple infections increase with decreasing CD4 count and increasing HIV viral load. We did not observe this trend; however, our small sample precludes the ability to make a meaningful comparison.

In contrast to other studies, we focused on high-grade lesions as opposed to mostly low grade lesions. The limitation of the other studies on HIV-infected women was that they used mostly cytological samples whose diagnoses were not always confirmed by histological review. The strategy employed in this study is more expensive and labor intensive than cytology alone, but also more precise. Histologically diagnosed CIN 3 is considered as a high-risk cervical cancer pre-cursor, and is an ideal source for HPV genotyping in terms of its high positive predictive value for progression to invasive cervical cancer as well as the fact that it is associated with the same HPV genotypes in the similar proportions as it is associated with cervical cancer. CIN 2 and lesser grade lesions have poor diagnostic reproducibility and have less positive predictive value than CIN 3 (17). Because the cytological diagnoses of HGSIL groups CIN 2 and CIN 3 together, the correlation of HPV types with HGSIL is not as precise as with histologically confirmed severe dysplasia or CIN 3/CIS. Therefore, strength of our study is that we used histologically confirmed CIN 3 lesions instead of cytology alone. Given the uncommon incidence of invasive cervical cancer in a HIV-positive population with access to health care such as our patient group (21), we felt that the inclusion of high-grade lesions would increase our sample size while providing appropriate HPV type prevalence estimates for high-grade dysplasia (and extrapolates for invasive cervical cancer) in this population (Table 5).

As seen in Figure 1, the prevalence of non-16/-18 HR-HPV types decreases as the grade of dysplasia increases while the prevalence of HPV-16/-18 increases with grade of dysplasia in the general population. In our sample, HPV-16 prevalence in high-grade lesions was not different than that of the general population. What was different was the relative increase in prevalence of other HR-HPV types as compared to the general population in which they usually decrease and are less prevalent than HPV-16/-18 in higher grade lesions. One explanation is that HPV-16 continues to be as carcinogenic in HIV with similar efficiency, but an advantage is conferred to the other less efficient types thereby increasing their prevalence in high-grade lesions. This advantage likely occurs because the decreased vigilance of the immune system in these women allows for less virulent strains to result in malignant transformation as compared to their immunocompetent counterparts in whom the more virulent strains such as HPV-16 prevail.

In summary, our study design was based on the positive predictive value of CIN 3 lesions in predicting HPV genotypes in invasive cervical cancer from the same population. Strength of our study was the use of histological pathology review for all samples confirming the grade of the lesions. Our findings are consistent with that of others who found rare HR-HPV types to be more common in dysplasia of HIV-infected women. In this pilot study, the sample was small (*n* = 23), however, these preliminary results support the feasibility of retrospective HPV genotyping from archival paraffin-embedded blocks in our lab as well as the need for further research in this area. Because developing countries are known to have higher rates of HIV than the developed world and HPV is more aggressive in HIV-infected individuals, the highest potential for global public health impact by a HPV vaccine would be conferred by its effectiveness in the HIV-infected population or those at risk of acquiring the infection. We are now planning to better characterize differences between HIV-infected and HIV-negative women in terms of their HPV type prevalence and their immunity profiles with the goal of guiding the development of both therapeutic and prophylactic vaccines that would be effective not only in the general population but also in the HIV-infected population.

## Conflict of Interest Statement

The authors declare that the research was conducted in the absence of any commercial or financial relationships that could be construed as a potential conflict of interest.
